# PFOS Inhibited Normal Functional Development of Placenta Cells via *PPARγ* Signaling

**DOI:** 10.3390/biomedicines9060677

**Published:** 2021-06-15

**Authors:** Jing Li, Xiaojie Quan, Saifei Lei, Zhenyao Huang, Qi Wang, Pengfei Xu

**Affiliations:** 1 School of Public Health, Xuzhou Medical University, Xuzhou 221002, China; xzmc2009@gmail.com (J.L.); quanxiaojie7@gmail.com (X.Q.); huangzhenyao@gmail.com (Z.H.); wangqixzmu2018@163.com (Q.W.); 2 Center for Pharmacogenetics, Department of Pharmaceutical Sciences, University of Pittsburgh, Pittsburgh, PA 15261, USA; sal208@pitt.edu; 3 Beijing Key Laboratory of Gene Resource and Molecular Development, College of Life Sciences, Beijing Normal University, Beijing 100875, China

**Keywords:** perfluorooctane sulfonic acid, placenta, *PPARγ*, cell growth, cell migration

## Abstract

Perfluorooctane sulfonic acid (PFOS), a persistent environmental pollutant, has adverse effects on gestation pregnancy. Peroxisome proliferator-activated receptor γ (*PPARγ*) is involved in angiogenesis, metabolic processes, anti-inflammatory, and reproductive development. However, the function of *PPARγ* in PFOS evoked disadvantageous effects on the placenta remain uncertain. Here, we explored the role of *PPARγ* in PFOS-induced placental toxicity. Cell viability, cell migration, angiogenesis, and mRNA expression were monitored by CCK-8 assay, wound healing assay, tube formation assay, and real-time PCR, respectively. Activation and overexpression of *PPARγ* were conducted by rosiglitazone or pcDNA-*PPARγ*, and inhibition and knockdown of *PPARγ* were performed by GW9662 or *si-PPARγ*. Results revealed that PFOS decreased cell growth, migration, angiogenesis, and increased inflammation in human HTR-8/SVneo and JEG-3 cells. Placenta diameter and fetal weight decreased in mice treated with PFOS (12.5 mg/kg). In addition, rosiglitazone or pcDNA-*PPARγ* rescued cell proliferation, migration, angiogenesis, and decreased inflammation induced by PFOS in HTR8/SVneo and JEG-3 cells. Furthermore, GW9662 or *si-PPARγ* exacerbated the inhibition of cell viability, migration, angiogenesis, and aggravated inflammation induced by PFOS in HTR-8/SVneo and JEG-3 cells. Meanwhile, the results of mRNA expression level were consistent with the cell representation. In conclusion, our findings revealed that PFOS induced placenta cell toxicity and functional damage through *PPARγ* pathway.

## 1. Introduction

Endocrine-disrupting chemicals (EDCs) are one out of a multitude of chemicals that can impair normal human development by altering homeostasis through the action of endogenous hormones or other endocrine signaling substances [1]. Perfluorooctane sulphonate (PFOS) is one of the most abundant perfluorinated chemicals that can accumulate biologically and be transported through all environmental media [2]. It has been reported that PFOS can be absorbed through several routes, including ingestion, absorption, and inhalation from food, drinking water, consumer goods, dust, aerosols, and chemical manufacturing facilities [3]. Based on a worldwide human biomonitoring study, geometric mean (GM) concentrations of PFOS range from 3.0 to 29.0 ng/mL in blood, 1.1 to 11.0 ng/mL in human cord blood, and 0.06 to 0.18 ng/mL in breast milk [4]. A British study reported that PFOS was detected in maternal serum samples during pregnancy, and median concentrations (interquartile range) were 13.8 (11.0, 17.7) ng/mL. High levels of PFOS (13.8 ng/mL) in prenatal maternal (30 weeks) serum may be associated with reduced weight of male infants at birth [5]. Studies in pregnant mice found that PFOS can pass through the placental barrier and induce developmental toxicity, such as in prenatal mortality and fetal growth retardation [6,7]. Our previous research has also shown that PFOS can reduce birth weight and damage the placenta in mice [8]. However, the mechanisms of PFOS-induced fetal developmental toxicity remain unclear.

The peroxisome proliferator-activated receptors (PPARs) family is composed of PPARα, PPARβ/PPARδ, and *PPARγ* [9]. PPAR consists of five modular domains with domain E mediated ligand dependent transcriptional activation, which induces conformational changes in these receptors, leading to the recruitment of cofactor/co-activator proteins and subsequent heterodimerization of these receptors with retinoid X receptor (RXR) [10]. PPARs are ligand-inducible transcription factors that play crucial roles in angiogenesis, metabolic, anti-inflammatory, reproductive developmental processes, and regulate the expression level of plural genes such as *VEGFA* and *TNF-α* [11,12,13]. In terms of the PPAR subtypes, *PPARγ* is primarily involved in placental development. It is a critical component of trophoblastic differentiation and essential for trophoblastic maturation to establish maternal fetal transport [14,15]. Moreover, the dysfunctions of *PPARγ* in trophoblast cause several diseases associated with pregnancy, including recurrent miscarriage, intrauterine growth restriction (IUGR), preeclampsia (PE), and gestational diabetes mellitus (GDM) [16]. PPARγ has been illustrated as a master regulator to activate the transcription of multiple genes associated with cell migration, proliferation, and angiogenesis, such as *VEGFA*, *ANGPTL4*, *MMP-2*, and *MMP-9* [17,18]. In addition, substantial studies of mouse knockout models have described a massive placental defect that can be reversed by restoring the *PPARγ* gene via chimeras, revealing that *PPARγ* was essential for normal placental development in the mouse and homozygous *PPARγ* deficient mice embryos died due to placental dysfunction [14,19]. In particular, the deletion of *PPARγ* gene disrupts the terminal differentiation of trophoblast and placental vascularization [20].

PFOS is known as an activator of PPARs, primarily PPARα and *PPARγ* [21]. Research has reported that PFOS mediates renal tubular cell apoptosis through activation of *PPARγ* [22]. In addition, activation of *PPARγ* rescued PFOS induced proliferation inhibition in rat primary embryonic neural stem cells [23]. However, little is known about whether *PPARγ* is involved in the placental toxicity of PFOS. Here, we proposed to elucidate whether *PPARγ* plays a role in placental toxicity induced by PFOS and whether its mechanism is responsible for disrupting placental function.

## 2. Materials and Methods

### 2.1. Reagents

PFOS (potassium salt; >98% pure) was purchased from MAYA-R (Jiaxing, China). Dimethyl sulfoxide (DMSO) and the cell counting kit-8 (CCK-8) were purchased from Vicmed (Busan, Korea). MEM medium was purchased from Corning (Shanghai, China). DMEM/F12 medium and RPMI 1640 medium were purchased from KeyGEN BioTECH (Nanjing, China). Fetal bovine serum was purchased from ExCell Bio. HiScript^®^ II Q RT SuperMix for RT-PCR and AceQ^®^ RT-PCR were obtained from Vazyme (Nanjing, China). Rosiglitazone and GW9662 were purchased from MedChemExpress (Shanghai, China). pcDNA-*PPARγ* and siRNAs were obtained or synthesized by GenePharma (Shanghai, China). Lipofectamine 2000 reagent (Invitrogen, Carlsbad, CA, USA) was purchased from Invitrogen and used for transient transfection.

### 2.2. Cell Culture and Animal Treatment

The human choriocarcinoma cell line HTR-8/SVneo and JEG-3 cells were a gift from Nanjing Medical University (Nanjing, China) and cultured in MEM medium supplemented with 10% heat-inactivated FBS and grown in 5% CO_2_ at a 37 °C humidified incubator. Animals were treated according to the guidance for the Care and Use of Laboratory Animals released by the US National Institute of Health. Animal experiments and procedures were approved by both the local animal care committee and the Animal Ethics Committee of Xuzhou Medical University (protocol 201605w025, 25 May 2016). 10 week old female and male mice with weights of 30–35 g, were chosen in our research. All of the mice were placed in a 12 h light and 12 h dark cycles and accessed food with water freely. Vaginal plug appearance was observed at day 0.5 of gestation (GD0.5). Two females were mated with one male overnight, and the presence of a vaginal plug was defined as gestational day (GD) 0. Pregnant mice were randomly divided into three groups of eight and were orally administered with 0, 0.5, 2.5, and 12.5 mg/kg/day PFOS from GD1 to GD17. The corn oil (10 mL/kg) treated the same as controls. Euthanization was then performed on GD18, and laparotomies were performed for pregnant mice. The placenta samples were promptly frozen in liquid nitrogen and stored at −80 °C.

### 2.3. si-PPARγ and pcDNA-PPARγ Transfection

The *si-PPARγ* (50 nM), *si-Control* (50 nM) (GenePharma, Shanghai, China), pcDNA-*PPARγ* (2 μg), and pcDNA 3.1 (2 μg) (GenePharma, Shanghai, China) were transfected using the Lipofectamine 2000 reagent in six-well culture plates. To achieve *PPARγ* knockdown and overexpression in HTR-8/SVneo and JEG-3, cells were cultured in six-well plates for 24 h and after 4 h of transfection, DMEM/F12 and MEM supplemented with 10% FBS was added for 24 h.

### 2.4. Cell Viability Assay

Cell proliferation rates were subsequently evaluated using the CCK-8 in accordance with the manufacturer’s instructions. A total of 10 μL of the CCK-8 solution reagent was pipetted into each well of the 96-well plate with 100 μL of culture medium. The absorbance at 450 nm was detected using a microplate reader.

### 2.5. Cell Migration Assay

Cells were cultured in six-well plates until confluence. After incubation, an artificial wound of scratched cells was made by a 10 μL pipette tip and three scratches along the wound were marked randomly, then rinsed with PBS and cultured with serum-free medium for 24 h. The distances migrated by the cells were calculated by subtracting the distances of the wound at 24 h from that of the 0 h time point. Analysis of the wound healing distances was conducted by using the Image J software.

### 2.6. Tube Formation Assay

The tube formation assay was performed as previously described [24]. HTR-8/SVneo and JEG-3 cells were seeded at 2 × 10^5^ cells/well. The wells of a 96-well plate were filled with 50 µL of Matrigel (BD Biosciences, San Jose, CA, USA) incubated at 37 °C for 45 min to form gels and seeding HUVEC 5 × 10^3^ cells/well for 4 h and incubator in 5% CO_2_ at 37 °C. Appropriate images were acquired by fluorescence microscopy, three random microscopic fields were seeded per repeat well. The magnification of all the micrographs is 100×. The key parameters were total tube branch length, then quantified by Image J Software (v1.8.0).

### 2.7. Real-Time PCR (RT-PCR)

Total RNA was extracted using TRIzol (Invitrogen, Carlsbad, CA, USA) according to the manufacturer’s instructions. RNA reverse transcription kit was obtained from Vazyme (Nanjing, China). Firstly, the RNA template was briefly treated with 4 × GDNA wiper Mix to remove genomic DNA contamination. Subsequent direct addition of 5 × qRT Supermix II resulted in immediate reverse transcription. 500 ng RNA was then reverse transcribed. Quantitative real-time PCR was performed using the SYBR Green qPCR SuperMix. *GAPDH* was selected as an internal control. All the procedures were conducted in accordance with the instructions of the manufacturer. The relative gene expression levels were calculated by the 2^−ΔΔCt^ method. Specific primer sequences used in this study were obtained from Invitrogen Bioengineering Corporation (Shanghai, China) and listed in Table S1.

### 2.8. Statistical Analysis

All of the assay was repeated at least 3 times and all data were expressed as the mean ± SEM. Statistical analysis was performed using the SPSS 22.0 (IBM, Armonk, NY, USA). Differences between two groups were analyzed using Student’s *t*-test. The difference among multiple groups was assessed by one-way analysis of variance (ANOVA). Dunnett’s *t*-test was used for multiple comparisons with controls. GraphPad Prism software (version 8.0, San Diego, CA, USA), was used for data analysis and plotting. *p* < 0.05 was considered to be statistically significant.

## 3. Results

### 3.1. PPARγ Mediates PFOS-Induced Inhibition of Trophoblast Cells Survival and Proliferation In Vitro

**Figure 1:**
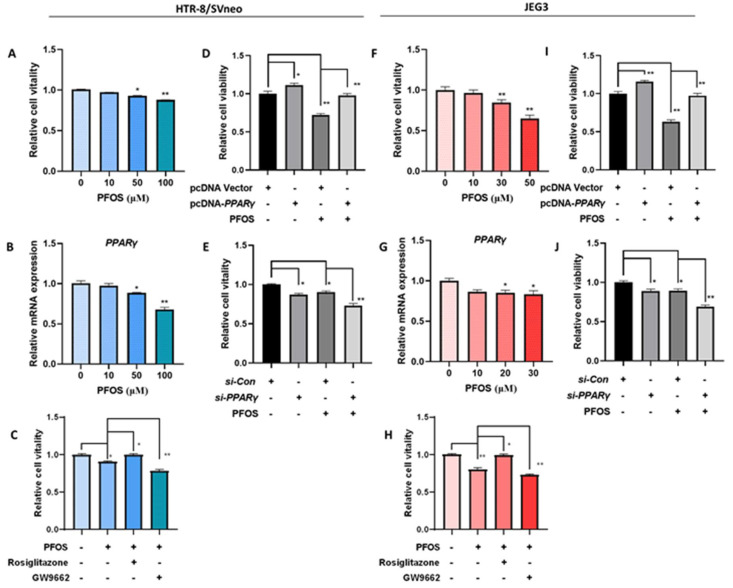
Effect of *PPARγ* on proliferation exposed to PFOS in vitro (50 μM for HTR-8/SVneo, 30μM for JEG-3). Cell vitality was detected by CCK-8 assay in (**A**) and JEG-3 (**F**) cells exposed to PFOS for 24 h. Relative mRNA expression levels of *PPARγ* were analyzed by RT-PCR in the two cell lines (**B**,**G**) exposed to PFOS for 24 h. Cell growth of HTR-8/SVneo (**C**) and JEG-3 cells (**H**) were then checked with PFOS treatment in the absence or presence of rosiglitazone and GW9662, and also estimated with treatment of PFOS when *PPARγ* was overexpressed and knocked down in HTR-8/SVneo (**D**,**I**) and JEG-3 cells (**E**,**J**). The data are shown as the means ± S.E.M. * *p* < 0.05; ** *p* < 0.01; compared with the indicated group, *n* = 3.

To investigate the effect of PFOS on cell viability, HTR-8/SVneo and JEG-3 cells were treated with different concentrations of PFOS. As shown in Figure 1A,F, HTR-8/SVneo and JEG-3 cells viability decreased gradually along with increased concentration of PFOS. PFOS significantly inhibited cell viability of HTR-8/SVneo cell and JEG-3 cells at 50 μM and 30 μM respectively. At these doses, PFOS significantly inhibited *PPARγ* mRNA expression levels in the two cell lines (Figure 1B,G). Rosiglitazone (a *PPARγ* agonist) and GW9662 (a *PPARγ* antagonist) were selected to explore whether *PPARγ* played a role in placental toxicity induced by PFOS. Results showed that rosiglitazone could partially rescue PFOS induced cell growth inhibition while GW9662 aggravated PFOS-induced cell growth inhibition significantly in HTR-8/SVneo and JEG-3 cells (Figure 1C,H), rosiglitazone and GW9662 were not toxic to cells at a range of concentrations (Figure S1A,B), and have little effect on the mRNA expression of *PPARγ* during PFOS treatment (Figure S2A,B).

**Figure 2:**
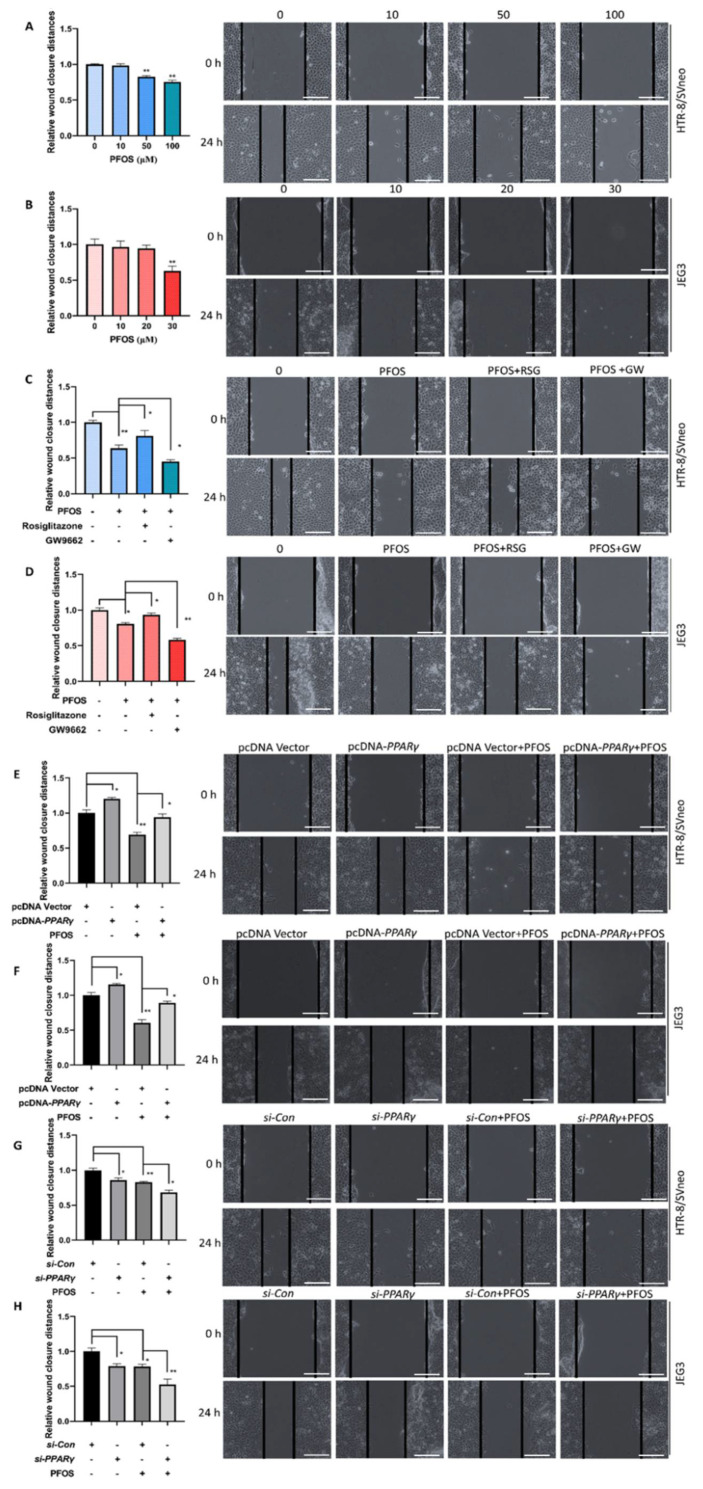
Effect of *PPARγ* on migration exposed to PFOS in vitro. The distances of the wound healing were measured after exposure of PFOS at 0 h and 24 h (**A**,**B**), exposure of PFOS in the absence or presence of rosiglitazone and GW9662 (**C**,**D**), with *PPARγ* overexpression (**E**,**F**) and knocking down (**G**,**H**) in HTR-8/SVneo and JEG-3 cells (50 μM for HTR-8/SVneo, 30 μM for JEG-3. Scale bar: 200 µm). The data are shown as the means ± S.E.M. * *p* < 0.05; ** *p* < 0.01; compared with the indicated group, *n* = 3.

Furthermore, findings showed *PPARγ* was overexpressed or knocked down in HTR-8/SVneo and JEG-3 cells, increased expression of *PPARγ* in the pcDNA-*PPARγ* group Figures S1C,D and S2C,D), and decreased expression in the *si-PPARγ* group Figure 1E,F and Figure 2E,F), validated by comparing to the control. Similar to *PPARγ* agonist rosiglitazone treatment, *PPARγ* overexpression could partially rescue PFOS-induced cell growth inhibition (Figure 1D,I). *PPARγ* knockdown significantly aggravated PFOS-induced cell growth inhibition in the cells as *PPARγ* antagonist GW9662 (Figure 1E,J).

### 3.2. PPARγ Is Important for Inhibition Effect of PFOS on the Cell Migration

PFOS dose-dependently induced cell migration was evaluated. The results revealed that PFOS remarkably decreased cell migration in HTR-8/SVneo cells at 50 μM (Figure 2A) and JEG-3 cells at 30 μM after 24 h treatment (Figure 2B). To elucidate the role of *PPARγ* in PFOS-induced trophoblast cell migration, wound healing assays were executed in the two cell lines pretreated with rosiglitazone or GW9662 and then co-treated with PFOS for 24 h. Rosiglitazone could partially alleviate PFOS-induced cell migration inhibition, whereas GW9662 could facilitate PFOS-induced cell migration inhibition (Figure 2C,D). Results also showed *PPARγ* overexpression alleviated cell migration inhibition (Figure 2E,F), but *PPARγ* knockdown aggravated cell migration inhibition by PFOS at 50 μM (Figure 2G) and 30 μM (Figure 2H) in HTR-8/SVneo and JEG-3 cells, respectively.

### 3.3. PPARγ Is Involved in Impaired PFOS–Induced Angiogenesis

**Figure 3:**
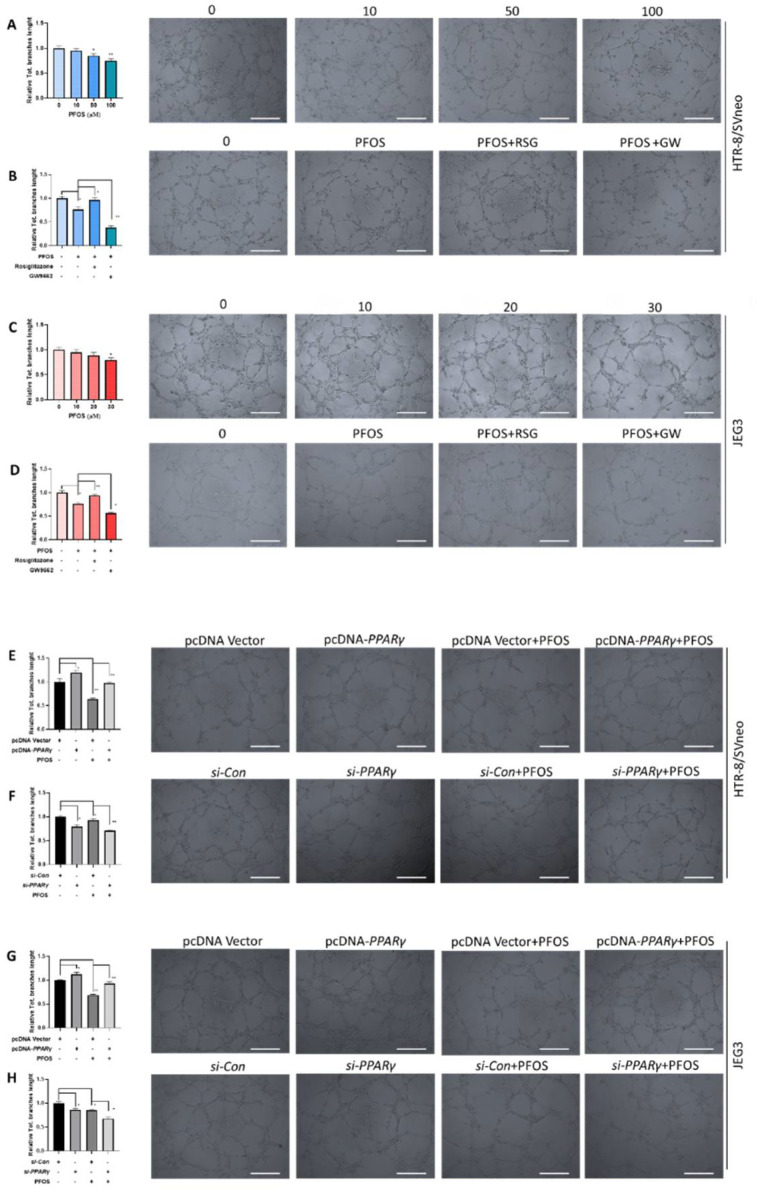
Effect of *PPARγ* on angiogenesis exposed to PFOS in vitro. The branches length was measured in HTR-8/SVneo and JEG-3 cells exposed to PFOS for 24 h (**A**,**C**), in the absence or presence of rosiglitazone and GW9662 (**B**,**D**), with *PPARγ* overexpression (**E**,**G**) and knocking down (**F**,**H**) in HTR-8/SVneo and JEG-3 cells (Scale bar: 200 µm). The data are shown as the means ± S.E.M. * *p* < 0.05; ** *p* < 0.01; compared with the indicated group, *n* = 3.

To explore the role of *PPARγ* in PFOS-induced angiogenesis, tube formation assay was performed in HUVECs (Human Umbilical Vein Endothelial Cell) with co-treatment of rosiglitazone or GW9662 and PFOS for 24 h. PFOS exposure suppressed angiogenesis in both HTR-8/SVneo (50 μM) and JEG-3 cells (30 μM) (Figure 3A,C), co-treatment of rosiglitazone rescued angiogenesis inhibition of PFOS and significantly increased the total tube branch length. Co-treatment of GW9662 and PFOS significantly decreased the total tube branch length compared to PFOS group in those cell lines (Figure 3B,D). Moreover, *PPARγ* overexpression alleviated angiogenesis inhibition induced by PFOS (Figure 3E,G), while *PPARγ* knockdown aggravated angiogenesis inhibition induced by PFOS (Figure 3F,H). Overall, these data suggested that *PPARγ* was an important mediator of PFOS-induced angiogenesis inhibition.

### 3.4. PFOS Alters mRNA Level of PPARγ Target Genes Associated with Proliferation and Angiogenesis

**Figure 4:**
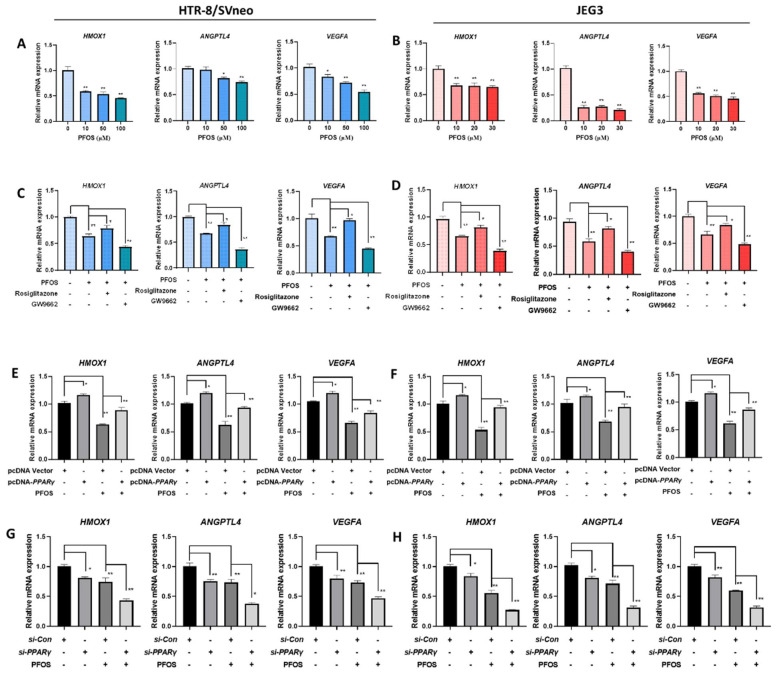
Effect of PFOS on *PPARγ* target genes associated with proliferation and angiogenesis in vitro. The mRNA expression was analyzed by RT-PCR in HTR-8/SVneo and JEG-3 cells were exposed to PFOS for 24 h (**A**,**B**), in the absence or presence of rosiglitazone and GW9662 (**C**,**D**), with *PPARγ* overexpression (**E**,**F**) and knocking down (**G**,**H**) in HTR-8/SVneo and JEG-3 cells. The data are shown as the means ± S.E.M. * *p* < 0.05; ** *p* < 0.01; compared with the indicated group, *n* = 3.

To understand the mechanisms of *PPARγ* in PFOS-induced effects on cell proliferation inhibition, migration inhibition, angiogenesis inhibition, pro-inflammatory in the two human placenta cell lines, we detected the expression of cell proliferation and angiogenesis related *PPARγ* target genes *HMOX1*, *ANGPTL4* and *VEGFA*. As shown in Figure 4A,B, *HMOX1*, *ANGPTL4*, and *VEGFA* mRNA expression were significantly reduced by PFOS exposure in HTR-8/SVneo (50 μM) and JEG-3 cells (30 μM). Co-exposure of PFOS and rosiglitazone up-regulated *HMOX1*, *ANGPTL4,* and *VEGFA* mRNA whereas co-treatment of GW9662 and PFOS significantly down-regulated these mRNA expressions (Figure 4C,D). Similarly, when HTR-8/SVneo and JEG-3 cells were co-treated with PFOS and overexpressed with *PPARγ*, *HMOX1*, *ANGPTL4,* and *VEGFA* mRNA expressions were significantly increased (Figure 4E,F). Knocking down of *PPARγ* and PFOS treatment significantly decreased *HMOX1*, *ANGPTL4,* and *VEGFA* mRNA expression levels (Figure 4G,H).

### 3.5. PFOS Alters mRNA Level of PPARγ Target Genes Associated with Migration

**Figure 5:**
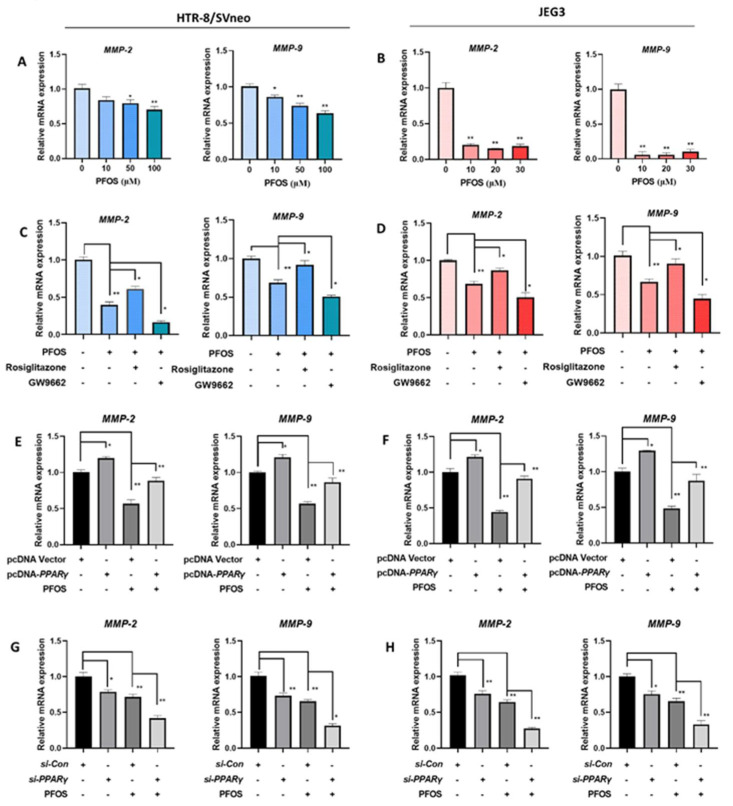
PFOS alters mRNA level of *PPARγ* target genes associated with migration. The mRNA expression was analyzed by RT-PCR in HTR-8/SVneoand JEG-3 cells were exposed to PFOS for 24 h (**A**,**B**), in the absence or presence of rosiglitazone and GW9662 (**C**,**D**), with *PPARγ* overexpression (**E**,**F**) and knocking down (**G**,**H**) in HTR-8/SVneo and JEG-3 cells. The data are shown as the means ± S.E.M. * *p* < 0.05; ** *p* < 0.01; compared with the indicated group, *n* = 3.

The expression levels of cell migration related *PPARγ* target genes *MMP-2* and *MMP-9* were detected. *MMP-2* and *MMP-9* mRNA expression were significantly decreased in PFOS-exposed groups (Figure 5A,B). When HTR-8/SVneo and JEG-3 cells were exposed to PFOS and co-treated with rosiglitazone for 24 h, they were significantly increased, whereas with co-treatment of GW9662 and PFOS they were significantly decreased (Figure 5C,D). *MMP-2* and *MMP-9* mRNA expression levels were also significantly lifted when cells were treated with PFOS and overexpressed with *PPARγ* (Figure 5E,F), and significantly lowered with PFOS and transfected with *si-PPARγ* (Figure 5G,H).

### 3.6. PFOS Alters mRNA Level of PPARγ Target Genes Associated with Inflammation

**Figure 6:**
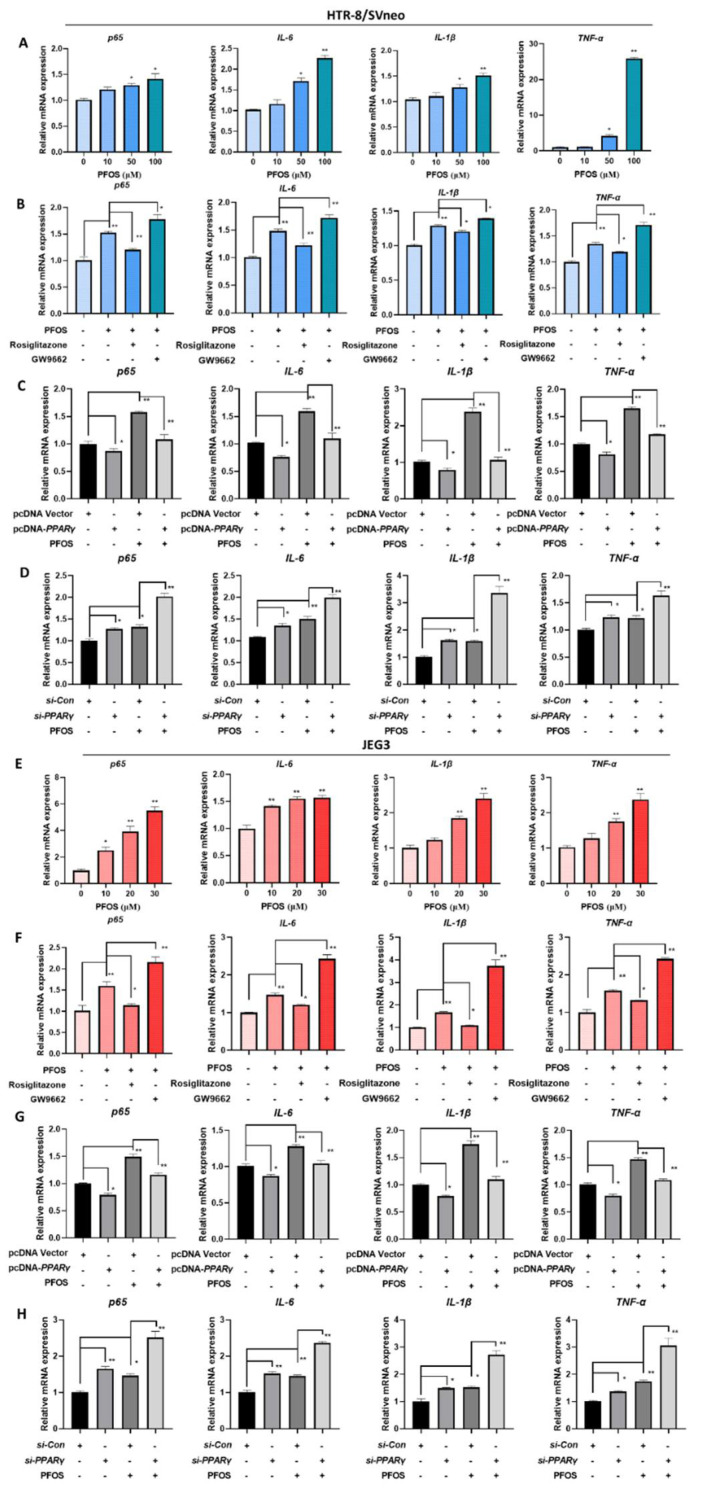
PFOS alters mRNA level of *PPARγ* target genes associated with inflammatory cytokines. The mRNA expression was analyzed by RT-PCR in HTR-8/SVneo and JEG-3 cells were exposed to PFOS for 24 h (**A**,**E**), in the absence or presence of rosiglitazone and GW9662 (**B**,**F**), with *PPARγ* overexpression (**C**,**G**) and knocking down (**D**,**H**) in HTR-8/SVneo and JEG-3 cells. The data are shown as the means ± S.E.M. * *p* < 0.05; ** *p* < 0.01; compared with the indicated group, *n* = 3.

The mRNA expression of *p65*, *IL-6*, *IL-1β,* and *TNF-α* were significantly increased with PFOS exposure in HTR-8/SVneo (50 μM) and JEG-3 cells (30 μM) (Figure 6A,E). With supplemented exposure of rosiglitazone for 24 h, the expression levels of those genes were significantly decreased compared to PFOS groups although increased when exposed to GW9662 for 24 h (Figure 6B,F). Consistently, when cells were treated with PFOS and *PPARγ* over-expressed, *p65*, *IL-6*, *IL-1β,* and *TNF-α* mRNA expressions were decreased (Figure 6C,G), levels of those genes were raised up when cells were treated with PFOS and *PPARγ* knocked down at the same time (Figure 6D,H).

### 3.7. PFOS Induces Placenta Dysfunction in Mice

**Figure 7:**
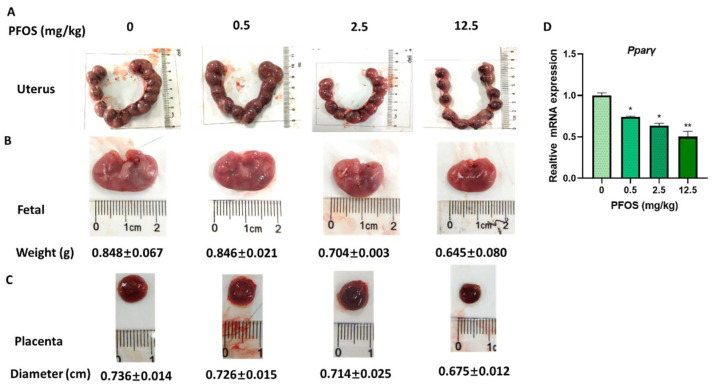
PFOS exposure causes developmental toxicity in placental (**A**) Representative picture of uterus in GD17 mice. (**B**) Representative picture of fetus. (**C**) Representative picture of placenta. (**D**) The mRNA expression was analyzed by RT-PCR in GD17 mice placental. The data are shown as the means ± S.E.M. * *p* < 0.05; ** *p* < 0.01; compared with control group, *n* = 3.

To determine the toxicity of PFOS to placenta in vivo, uterus, placenta size, and fetal weight were detected in PFOS-exposed mice. Our results showed that placental diameter and fetal weight decreased in the PFOS-treated mice compared with controls (Figure 7A–C). Our previous research indicated that the relative number of Ki67 positive cells reduced placental angiogenesis of PFOS-treated mice, which suggested PFOS might affect placental angiogenesis by inhibiting the proliferation of vascular cells [25].

### 3.8. PFOS Alters PPARγ Target Genes mRNA Expression in Mice Placenta

**Figure 8:**
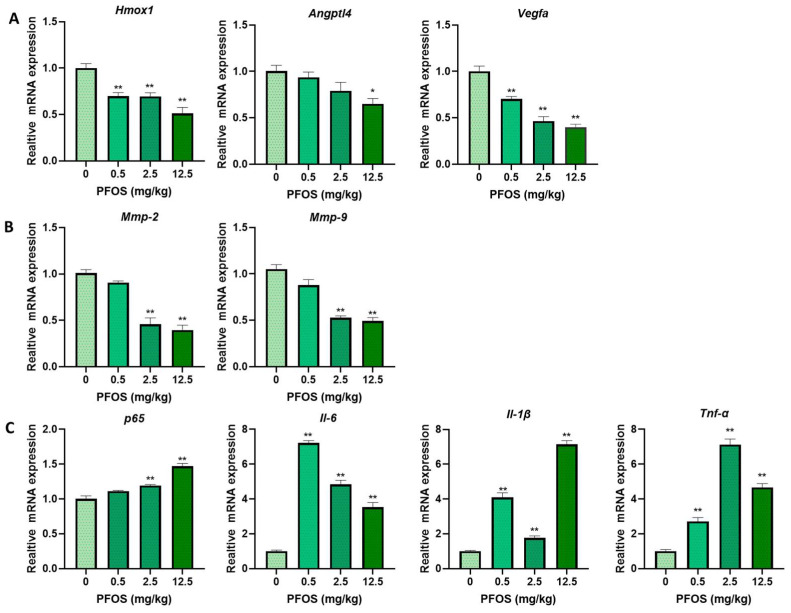
Gestational PFOS exposure alters mRNA level of *PPARγ* target genes in mouse placentas. Relative expression levels of *PPARγ* target proliferation, angiogenesis (**A**), migration (**B**), and inflammation (**C**) related genes in placentas of mice were analyzed by RT-PCR. The data are shown as the means ± S.E.M. * *p* < 0.05; ** *p* < 0.01; compared with control group, *n* = 3.

PFOS treatment decreased the mRNA expression of *PPARγ* in the placental tissues in dose-dependent settings (Figure 7D). The mRNA level of *PPARγ* target genes in the placental tissues of PFOS-treated mice were detected. The relative expression of *Homx1, Angptl4*, *Vegfa, Mmp-2*, and *Mmp-9* in placentas were decreased in the PFOS-exposed group (Figure 8A,B). Furthermore, the relative expression of *p65*, *Il-6*, *Il-1β,* and *Tnf-α* were increased in the placentas of PFOS-exposed mice (Figure 8C), which were all consistent with the results in vitro.

## 4. Discussion

Despite multiple developmental toxicities shown to be induced by PFOS, the mechanism of PFOS-elicited severe placental dysfunction has not been well investigated. Here, our research group investigated the mechanisms of PFOS-elicited effects on the function of trophoblast cells in vitro and in vivo. We demonstrated whether *PPARγ* is involved in the toxicity of PFOS by regulating placental cell growth, angiogenesis, and inflammatory responses in HTR-8/SVneo and JEG-3 cells. Our results indicated that PFOS dose-dependently inhibited cell growth in HTR-8/SVneo and JEG-3 cells, which corresponded to the discoveries of our previous study in mice [25]. Cell proliferation contributes significantly to placental growth during gestation [26]. The imbalance in human syncytiotrophoblast proliferation may contribute to multiple adverse pregnancy outcomes, such as miscarriage, preeclampsia, preterm birth, and fetal growth restriction [27]. Pham also found there to be undesirable reproductive complications associated with prenatal exposure to PFOS, including preeclampsia [28]. In addition, PFOS weakens the migration capacity of HTR-8/SVneo and JEG-3 cells. These effects could be associated with the decreased levels of MMP-2 and MMP-9, it is known that several cell migration gene decreases of above gene expression have been implicated in human placental dysfunction or pregnancy complication progression in several human pregnancies [29]. A previous study also reported that PFOS inhibited trophoblast migration and decreased the mRNA expression of MMPs involved in migration [30].

Angiogenesis is a biological approach that has formatted new vascular beds and is a critical process to provide tissue growth and development with oxygen and nutrients [31]. Placental angiogenesis seems to play an important role in the development of viable and healthy offspring [32]. Decreased placental vascular development and increased vascular resistance have been believed to be associated with early embryonic mortality [33,34]. Our results describe that PFOS dose-dependently inhibited angiogenesis in HTR-8/SVneo and JEG-3 cells, and our previous study showed that blood vessel branching was significantly reduced in the labyrinth layer of mice treated with PFOS [25]. Moreover, treatment with PFOS also reduced the expression of *PIGF,* a potent angiogenic factor, which is implicated in preeclampsia and IUGR [28]. In addition, PFOS inhibited *VEGFA* mRNA expression dose-dependently in HTR-8/SVneo and JEG-3 cells, which is a major angiogenic growth factor of the placenta [35].

*PPARγ* has also been reported to play a key role in placental development. *PPARγ* null mutant placentae accumulate lipid droplets in the labyrinth barrier, and failure of vascular labyrinth formation leads to vascular anomalies and major placental dysfunctions that in turn result in embryonic lethality [36]. *PPARγ* regulates differentiation, maturation, secretion, fusion, proliferation, migration, angiogenesis, and invasion of trophoblast cells by regulating lipid and glucose metabolism and inflammatory response [37]. To investigate the role of *PPARγ* on PFOS-induced inflammatory cytokines and inhibition of cell growth and angiogenesis, rosiglitazone, a specific agonist of *PPARγ*, was used to reverse PFOS-evoked downregulation of *PPARγ* pathway [38]. As expected, rosiglitazone significantly rescued PFOS-induced cell growth and angiogenesis inhibition in HTR-8/SVneo and JEG-3 cells. Previous studies have demonstrated that treatment of hypoxic JEG-3 cells with rosiglitazone improves cell survival and decreases apoptosis [39]. Treatment with rosiglitazone greatly increased wound healing and improved angiogenesis in mice with spontaneous glucose metabolic disorders [40]. For instance, rosiglitazone rescued *HO-1* expression and inhibited inflammation in myometrial and decidual macrophages in inflammation-induced preterm birth [41]. GW9662 is a potent *PPARγ* antagonist that prevents activation of *PPARγ* [38]. Pretreatment with GW9662 abolished cell migration and invasion in prostate cancer cells [42].

*PPARγ* downstream targets, such as *ANGPTL4*, *MMP-2*, *MMP-9*, *HO-1*, *VEGFA*, *p65*, *TNF-α*, *IL-6*, and *IL-1β* play essential roles in inhibiting cell migration, angiogenesis, and inflammation response [43,44]. Our gene expression data have demonstrated that PFOS induced dysfunction of HTR-8/SVneo and JEG-3 cells were characterized by an imbalance of cell proliferation, migration, angiogenic and inflammatory factors, in terms of increased *p65*, *TNF-α*, *IL-6*, and *IL-1β* levels and decreased *ANGPTL4*, *MMP-2, MMP-9*, *HO-1,* and *VEGFA* levels. *ANGPTL4*, as a transcription target of *PPARγ*, participated in cellular functional regulation including cell survival, proliferation, migration, and invasion in trophoblast cells [45]. *PPARγ* activation has been demonstrated that up-regulate HO-1 expression an antioxidant enzyme and decreased sFlt-1 the production of the anti-angiogenic mediator [46,47]. Furthermore, *PPARγ* agonists have been illustrated to restore proangiogenic factors and upregulate *HO-1* and *VEGFA* expression in vitro and in vivo [47]. *MMP-2* and *MMP-9*, as downstream target genes of *PPARγ*, have been improved that could accelerate the trophoblast migration [18]. In the present study, rosiglitazone altered cell proliferation, migration, angiogenesis, and inflammation factors in the PFOS- induced HTR-8/SVneo and JEG-3 cells. Previous studies have reported that *HO-1* is regulated by *PPARγ* agonists and that induction of *HO-1* can prevent *TNF-α* induced endothelial dysfunction in vitro, which indicated that rosiglitazone may be responsible for protecting the vascular system via *HO-1* and potentially anti-inflammatory mechanism [48]. Untimely inflammatory triggers that shift immunological balance towards activation can lead to adverse pregnancy outcomes, including preterm birth and miscarriage [49]. Several researchers have demonstrated that exposure to inflammatory stimuli could induce the secretion of *p65*, *IL-6*, *TNF-α*, and *IL-1β* pro-inflammatory cytokine and cell apoptosis in trophoblast cells [50,51]. In our study, the expression of several inflammatory cytokines including *p65*, *TNF-α*, *IL-6,* and *IL-1β* were upregulated in the placentas of mice and HTR-8/SVneo and JEG-3 cells exposed to PFOS. Consistent with other research, treatment with PFOS (1.0 mg/L) increased the mRNA expression of *p65*, *IL-6,* and *TNF-α* in HTR-8/Svneo cells compared with the control group [52]. Ji et al. found that rosiglitazone significantly inhibited LPS-induced cell apoptosis, and inflammation in HUVECs [53]. GW9662 partially aggravated PFOS-induced inflammatory cytokines in HTR-8/SVneo and JEG-3 cells. Further, rosiglitazone rescued premature delivery, reduced inflammation, and improved both placental and fetal weight in a mouse model of inflammation-induced preterm birth [41,54]. *PPARγ* seemed to affect the inflammation by interacting with p65 in HTR-8/SVneo [55]. In the present study, administration of the *PPARγ* agonist rosiglitazone ameliorated both cell proliferation and placenta angiogenesis dysfunction via *ANGPTL4*, *MMP-2*, *MMP-9*, *HO-1*, and *VEGFA* dependent pathway in HTR-8/SVneo and JEG-3 cells. These results suggest that PFOS inhibit normal functional development of placenta cells through *PPARγ* pathway, at least partially in vitro and in vivo.

## 5. Conclusions

We demonstrated that PFOS negatively alters normal functional development of placenta cells partially through PPAR signaling. This study provides a novel insight into PFOS-induced placental toxicity. In particular, it investigates the molecular mechanism for *PPARγ* in abnormal placental development.

## Data Availability

The data sets generated and/or analyzed during the current study are available from the corresponding author on reasonable request.

## Supplementary Materials

The following are available online at https://www.mdpi.com/article/10.3390/biomedicines9060677/s1, Figure S1: Effect of rosiglitazone and GW9662 on proliferation in human HTR 8/SVneo and JEG 3 cells, Figure S2: Effect of PFOS on PPAR mRNA expression in human HTR8/SVneo and JEG 3 cells, Table S1: RT-PCR primers for analysis.
