# Supplementary material for: PFOS Inhibited Normal Functional Development of Placenta Cells via PPARγ Signaling
Source: Biomedicines. 2021 Jun 15;9(6):677. doi: 10.3390/biomedicines9060677 (PMC8232579; doi:10.3390/biomedicines9060677)
Supplement: Supplementary file 1 [file biomedicines-09-00677-s001.zip › biomedicines-1222927-supplementary.pdf]

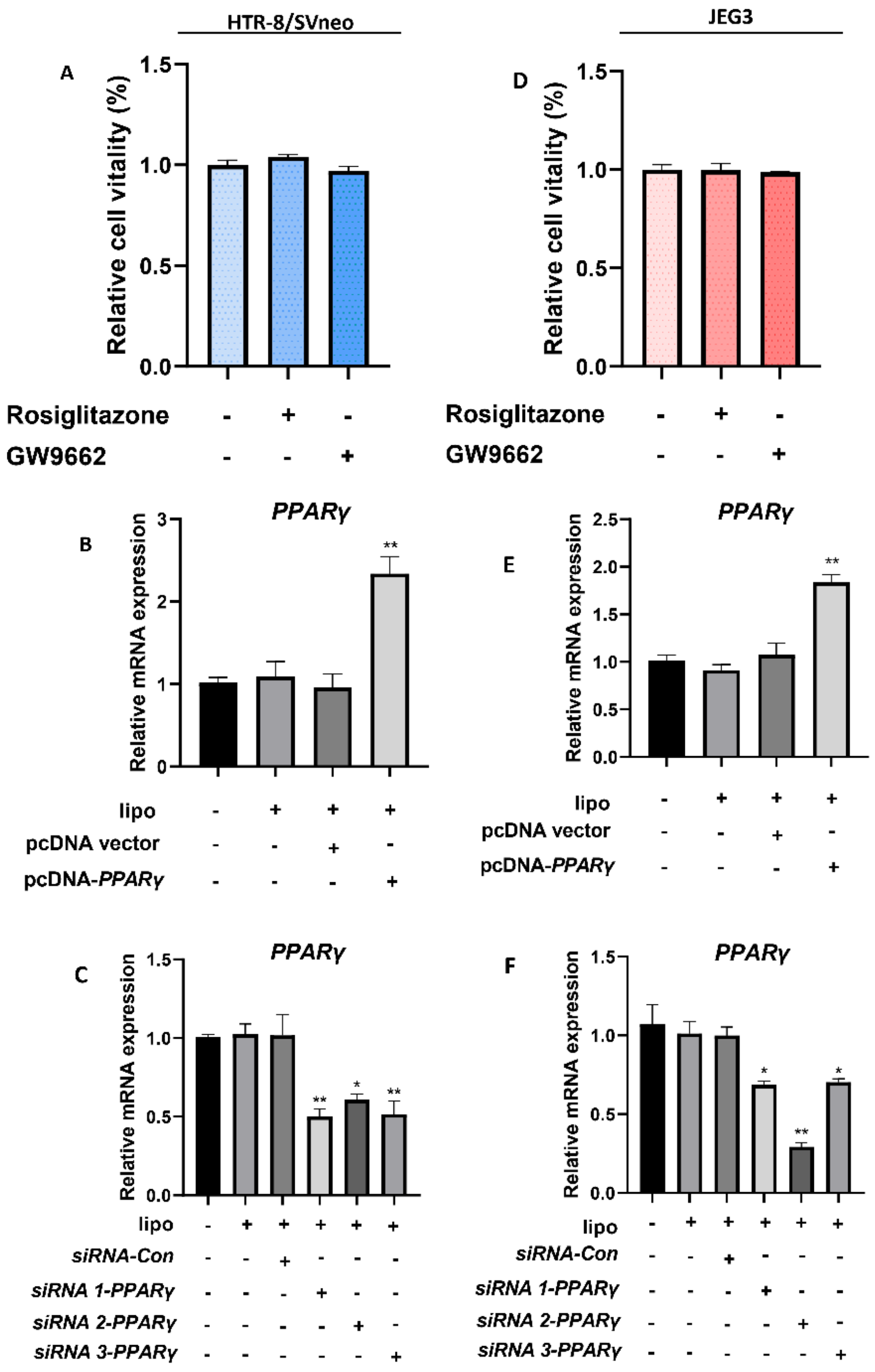

Figure S1. Effect of rosiglitazone and GW9662 on proliferation in human HTR-8/SVneo and JEG-3 cells. Cell growth was detected by CCK-8 assay in HTR-8/SVneo and JEG-3 cells treated with rosiglitazone and GW9662 for 24 h (A and B). Relative mRNA expression levels of *PPARγ* were validated by RT-PCR after the cells were *PPARγ* overexpressed (C and D) and knocked down (E and F). The data are shown as the means ± S.E.M. *$P < 0.05$; ** $P < 0.01$. n = 3.

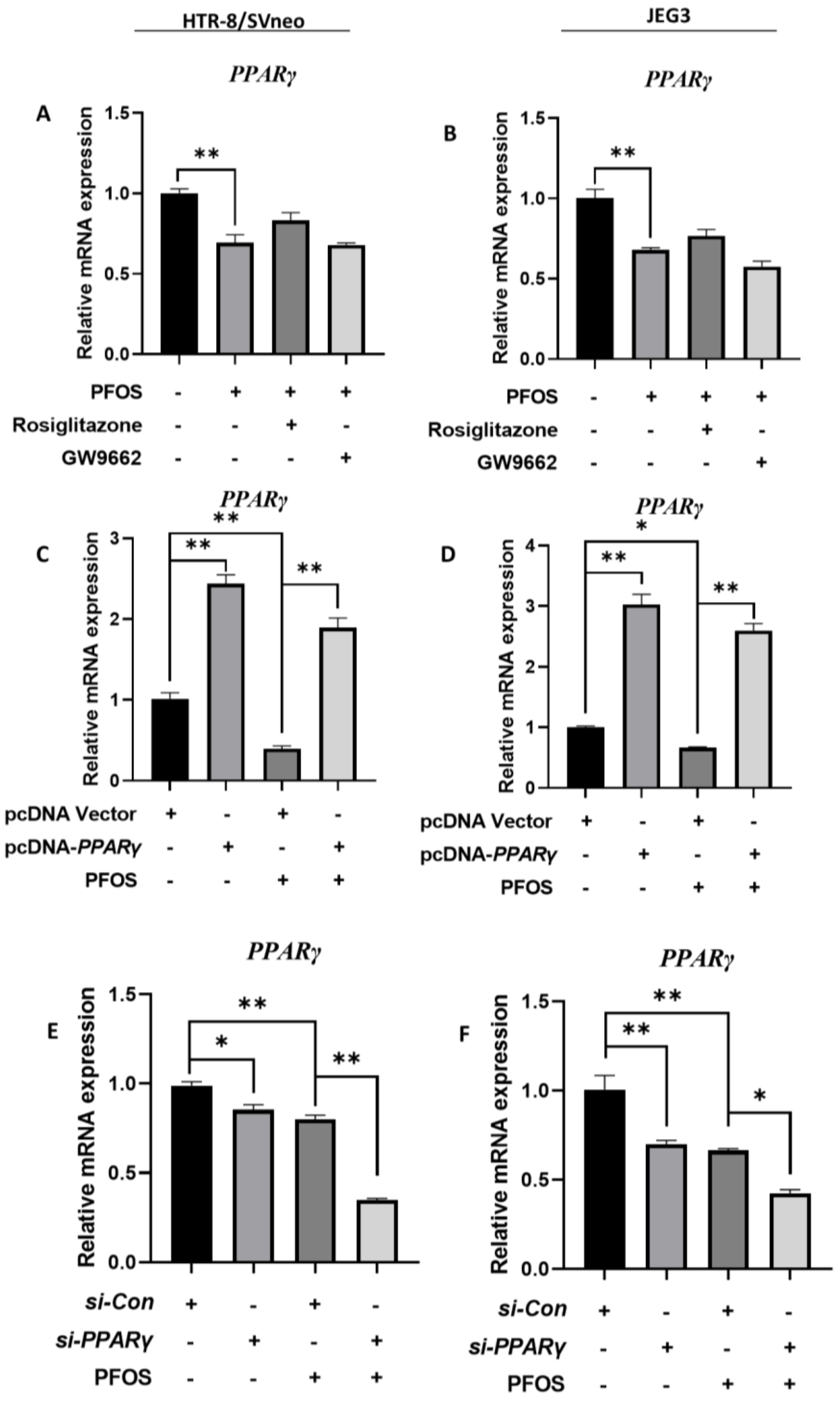

Figure S2. Effect of PFOS on PPAR mRNA expression in human HTR-8/SVneo and JEG-3 cells. Relative mRNA expression levels of *PPARγ* were validated by RT-PCR after HTR-8/SVneo and JEG-3 cells treated with rosiglitazone and GW9662 for 24 h (A and B). Relative mRNA expression levels of *PPARγ* were validated by RT-PCR after the cells were *PPARγ* overexpressed (C and D) and knocked down (E and F). The data are shown as the means ± S.E.M. $*P < 0.05$; $** P < 0.01$. n = 3.

Table S1. **RT-PCR primers for analysis**

Gene symbol	Forward primer (5' -> 3')	Reverse primer (5' -> 3')
hGAPDH	GGAAATCCCATCACCATCT	GGACTCCACGACGTACTCA
hANGPTL4	CTCAAGGCTCAGAACAGCAGG	TGGTCCAGGAGGCCAAACT
hHMOX1	CAGCGGGCCAGCAACAAAG	ACCCATCGGAGAAGCGGAGC
hVEGFA	AGGGCAGAATCATCACGAAG	GAAGATGTCCACCAGGGTCTC
hMMP-2	CTTCCAGGGCACATCCTAT	CCTTCTGAGTTCCCACCAA
hMMP-9	TCCCTGGAGACCTGAGAACC	GCCACCCGAGTGTAACCAT
hp65	GGGGACTACGACCTGAATGCT	GTCAAAGATGGGATGAGAAAGGA
hTNF-α	TGAAAGCATGATCCGGGACG	AGGCAGAAGAGCGTGGTGGC
hIL-6	CAAATTCGGTACATCCTCG	TTTCTGCCAGTGCCTCTTT
hIL-1β	ATGGCTTATTACAGTGGCA	GTAGTGGTGGTCGGAGATT
mGapdh	TCTTGGGCTACACTGAGGA	ATACCAGGAAATGAGCTTGA
mAngptl4	ATCACAGGGAACCGAGGAA	ATTGGAGCAATTTGGCATT
mHmox1	GGTGATGGCTTCCTTGTAC	AGACTGGGTTCTGCTTGTT
mVegfa	GCACCCACGACAGAAGGAG	TCAATCGGACGGCAGTAGC
mMmp-2	TGTCCCGAGACCGCTATGT	TTGCCCAGGAAAGTGAAGG
mMmp-9	ACAGCCAACTATGACCAGGAT	TTGCCCAGGAAGACGAAGG
mp65	TGCGATTCCGCTATAAATG	TTGGTGGTATCTGTGCTTCTC
mTnf-α	TCTCATTCCTGCTTGTGGC	GGAACTTCTCATCCCTTTGG
mIl-6	CTTCTTGGGACTGATGCTG	GGTCTGTTGGGAGTGGTAT
mIl-1β	TGAAGGGCTGCTTCCAAAC	GATGTGCTGCTGCGAGATT
